# A one-arm pilot trial of a telehealth CBT-based group intervention targeting transdiagnostic risk for emotional distress

**DOI:** 10.1371/journal.pone.0303131

**Published:** 2025-06-18

**Authors:** Sierra Flynt, Grace Y. Cho, Brandon Koscinski, Catherine Accorso, Ashley Knapp, Stephanie Gorka, Julie Suhr, Megan Austin, Nicholas P. Allan

**Affiliations:** 1 Department of Psychology, Ohio University, Athens, Ohio, United States of America; 2 Department of Psychological and Brain Sciences, University of Massachusetts, Amherst, Massachusetts, United States of America; 3 Feinberg School of Medicine, Northwestern University, Evanston, Illinois, United States of America; 4 Department of Psychiatry and Behavioral Health, Ohio State University, Columbus, Ohio, United States of America; 5 VA Center of Excellence for Suicide Prevention, VA Finger Lakes Health Care System, Canandaigua, New York, United States of America; The Chinese University of Hong Kong, HONG KONG

## Abstract

The COVID-19 pandemic had a significant impact on mental health, straining an already overburdened healthcare system and highlighting health inequity issues. To streamline treatment efforts, targeting transdiagnostic risk factors for symptoms of emotional distress through a modular, transdiagnostic approach via telehealth may help to expand access to mental health treatment. Three transdiagnostic risk factors for emotional distress disorders that emerged as important treatment targets during the pandemic are anxiety sensitivity (AS) (*i.e.,* fear of anxious arousal), intolerance of uncertainty (IU) (*i.e.,* distress when confronted with uncertainty), and loneliness. To target AS, IU, and loneliness, we completed a pilot feasibility, acceptability, and utility trial of Coping Crew, our group, telehealth-delivered transdiagnostic treatment protocol. The 17 participants (*M*_*age*_ = 22.00, *SD *= 4.46; 71% female) rated the intervention and study protocol as feasible to deliver and acceptable and useful to address intervention targets. Evidence was mixed regarding feasibility, acceptability, and usefulness of the mobile app: 94% of the participants (n = 16) completed at least one daily survey 80% of the time, but only 35% of the participants (n = 6) completed at least 80% of the mobile app surveys over the course of the intervention. Most participants rated use of the app as acceptable and relevant to psychological improvements made due to the intervention. Exploratory analyses suggest potential reductions in transdiagnostic risk factors at post-intervention, which were maintained at 1- and 3-month follow-up. Detailed effect size estimates are provided. However, these should be interpreted with caution due to the small sample size and exploratory nature of the study.

## Introduction

The COVID-19 pandemic not only led to harmful physical health outcomes, but also increased negative mental health outcomes. Since the pandemic began, the percentage of people who reported their anxiety as ‘high’ or ‘extremely high’ has quadrupled from 5% to 20%, and the self-reported prevalence of depression has doubled [1]. In 2021, approximately two-thirds of individuals in the United States were experiencing moderate or severe psychological distress including elevated anxiety [2,3], depression [4], and loneliness [5]. Further, as the pandemic recedes, it is expected that people will continue to seek mental health services at a higher rate than before the pandemic [1,6]. Thus, there is an immediate need for brief, modular telehealth interventions targeting symptoms related to emotional distress. A flexible, transdiagnostic treatment approach may be optimal for addressing shared risk factors underlying emotional distress, particularly in response to the profound impact of this unprecedented environmental stressor. Anxiety sensitivity (AS), characterized by fear of anxious arousal; intolerance of uncertainty (IU), reflecting distress in uncertain situations; and loneliness are three key transdiagnostic risk factors exacerbated by the pandemic and strong predictors of emotional distress beyond its immediate effects.

### Transdiagnostic risk factors

Emotional distress disorders encompass numerous mood and anxiety disorders that often share overlapping symptoms such as anxiety sensitivity and emotional avoidance [7,8]. Despite these shared symptom clusters and the commonality of comorbidity among mental disorders, disorder-specific cognitive-behavioral therapy (CBT) is widely used to treat emotional distress disorders. In a national survey of 43,093 adults receiving treatment for mental health conditions in the United States, researchers found that 80.3% had two or more diagnoses [9]. Treatments could be more efficient and effective if they focused on the core processes underlying multiple comorbid conditions [10]. Transdiagnostic risk factors have been implicated in the etiology of multiple disorders [11], and can directly influence the intensity of symptoms such and their development over time. Further, a growing body of literature demonstrates that not only can CBT reduce transdiagnostic risk factors, but reductions in these risk factors can lead to improvements in emotional distress disorder symptoms [12–15]. Consequently, using CBT to target transdiagnostic risk factors may have broad effects across multiple disorders. Three transdiagnostic risk factors with the capacity to reduce symptoms across emotional distress disorders include anxiety sensitivity, intolerance of uncertainty, and loneliness. We focus on these three risk factors due to their overarching influence and gained prominence due to the COVID-19 pandemic.

#### Anxiety sensitivity.

Anxiety sensitivity (AS) is the fear of bodily sensations associated with anxiety [16,17]. Lower-order dimensions of AS capture fears centered on physical, cognitive, and observable anxiety sensations and experiences. Elevated AS has been implicated as a risk factor for a wide range of maladaptive psychopathology, including anxiety disorders, depression, substance use and misuse, and suicidal urges [18–21]. Further, AS is positively associated with COVID-19-related worry, anxiety, functional impairment, and symptom severity [22] and predicts fear of COVID-19 [23] suggesting this will be an important elevated risk factor to target for future pandemics. Already, there is ample evidence that a brief AS intervention can reduce AS and associated psychopathology, including anxiety, depression, suicidal ideation, insomnia, and PTSD symptoms among others [14,18,24–26].

#### Intolerance of uncertainty.

Intolerance of uncertainty (IU) reflects “a dispositional characteristic that reflects an individual’s tendency to react negatively to uncertain situations, events, or outcomes, as well as the corresponding desire for certainty and predictability [27].” There is strong evidence of a higher-order IU factor and mixed evidence for two lower-order IU factors: prospective IU or discomfort when confronted with future uncertainty, and inhibitory IU or difficulty tolerating uncertainty in the moment [28,29]. IU is implicated in the onset of various psychiatric diagnoses, including generalized anxiety disorder (GAD [30]) and obsessive-compulsive disorder (OCD [31]). Changes in IU are related to increases in social anxiety, worry, and negative affect [32], along with changes in anxiety and depression symptoms across diagnostic categories [33]. Elevated IU during the COVID-19 pandemic has been identified as a risk factor for depression [5,34,35], fear and anxiety [36], and suicidal ideation [37]. IU is also positively associated with fear of COVID-19 [23], COVID-19 worry, sensitivity to COVID-19-related symptoms, and COVID-19-related interference in daily activities due to worry [37]. Already, there is emerging evidence that IU can be targeted in brief [38] and more intensive interventions [39,40].

#### Loneliness.

Loneliness is the subjective evaluation of one’s social relations as being inadequate or otherwise not fulfilling one’s social needs and desires [41–43]. Loneliness is conceptualized as a unidimensional construct [44] and has been linked to various psychiatric diagnoses and related behavioral risk factors including anxiety disorders, depression [5], and suicide attempts [45]. In a recent meta-analysis, loneliness was also associated with smoking, excessive drinking of alcohol, overeating, restricting food, as well as low levels of physical activity, paranoia, psychosis, elevated mental health symptom severity, poor recovery prognosis, poor interpersonal functioning, and increased mortality [43]. Loneliness has also been identified as a potential risk factor for depression symptom severity, psychological distress, and financial worries during COVID-19 [5]. Almost half (41%) of a Canadian sample indicated that social isolation has significantly negatively influenced their mental health [1]. Brief interventions targeting perceived burdensomeness and thwarted belongingness, two constructs reflecting biases in perceived social relations have provided preliminary evidence that loneliness could be reduced through a brief intervention [46,47]. Further, more intensive interventions have also proven successful in reducing loneliness [48].

### Development of a modular CBT for transdiagnostic risk factors exacerbated by the COVID-19 pandemic

Modular CBT treatments use CBT principles (e.g., cognitive distortions) to target an individual process or set of related processes underlying psychopathology [49]. In this framework, risk processes such as AS, IU, and loneliness—can be assigned to a client depending on their specific needs [50]. Even in a group setting, a modular approach allows for personalization, as participants are guided to focus on the skills most relevant to how their particular transdiagnostic risk factors manfest themselves. Group discussions offer structure and support and individualized skills practice address participants’ unique concerns. Modular CBT treatments, with their flexible and adaptable nature, provide an ideal framework for designing interventions that effectively target and address transdiagnostic risk factors implicated in various emotional distress disorders [51].

A central premise of CBT is that treatment gains are consolidated through out-of-session skills practice by the client [52,53]. However, unlike in-session activities, these activities have traditionally occurred without direct support, which can reduce engagement and gains from between-session activities [52]. The ubiquitous nature of smartphones and the general comfort with mobile apps provides an excellent platform to increase between-session client engagement. Generalization of CBT skills to situations outside of the therapy room requires repeated rehearsal of treatment techniques when patients are experiencing real distress [54]. Ecological momentary assessment (EMA), delivered via mobile devices, offers an opportunity to easily implement skills practice as-needed by providing a monitoring system that can determine when skills practice might be most beneficial. By using EMA data, ecological momentary interventions (EMIs) provide a framework for ideal learning by providing the type of precise support in real time and in real world settings [55]. The use of a mobile app also allows for accountability of completing behavioral exercises, another central component of most CBT-based treatments. In addition to improving the intervention, EMA also reduces retrospective recall bias, increases ecological validity, and allows for examination of short-term temporal dynamics [56].

### Current study

Brief, modular, transdiagnostic interventions based in empirically-supported CBT principles and delivered via a mobile app offer an opportunity to efficiently and effectively target several symptoms of emotional distress disorders exacerbated by the COVID-19 pandemic. AS, IU, and loneliness are three relevant risk factors to target given their associations with a wide range of psychopathology and distress due to the COVID-19 pandemic as well as evidence that they can be targeted through brief modules. We developed a 5-session group intervention, Coping Crew, using a modular framework and guided by CBT principles. A mobile app was used to track mood, provide brief cognitive nudges, and monitor interoceptive exposure (IE) and behavioral experiments (BEs) done as homework. The main purpose of the study was to examine the acceptability, feasibility, and utility of Coping Crew and the associated mobile app. Additionally, we explored preliminary effect size estimates and associations between transdiagnostic risk factors and emotional distress disorder symptom change over time.

## Methods

### Participants

Participants (*N* = 17) included 16 college students (94%) and one community member (6%) from a rural community in Southeast Ohio. Regarding self-reported race, 10 (59%) reported White, another two reported Asian and White (11.8%), another one reported Black (5.9%), and one reported another category (5.9%). All participants were non-Hispanic. Participants were 22 years old on average (standard deviation [*SD*] = 4.46), and 71% of participants were female.

### Procedures

**Fig 1:**
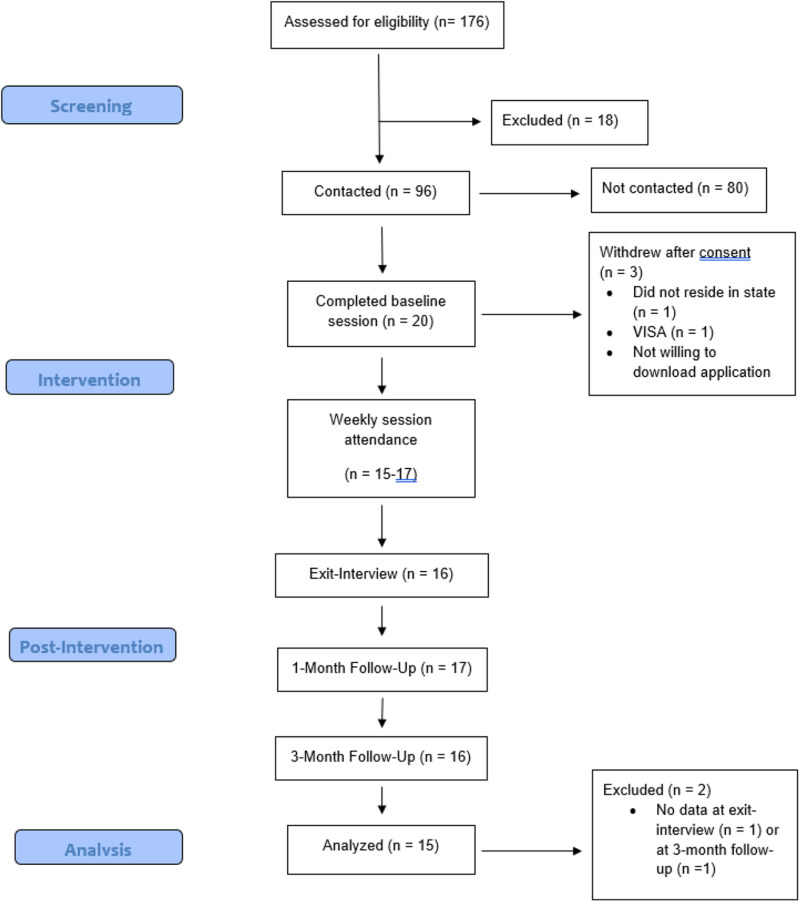
CONSORT diagram reflecting study recruitment and retention.

This study was supported by an internal grant provided to the last author and registered on clinicaltrials.gov (CT# NCT05019053). Participants were recruited through email, social media, and digital flyers to clients of the university psychology clinic. To be eligible, participants had to a) report either elevated AS (Anxiety Sensitivity Index-3 [ASI-3] ≥ 24), IU (Intolerance of Uncertainty Scale-12 [IUS-12] ≥ 35), or loneliness (NIH Toolbox Loneliness scale ≥ 13), b) own a smartphone, and c) have internet access. Exclusion criteria included current participation in other lab intervention studies (e.g., [57]), endorsement of psychotic features, or imminent risk for suicide. See Fig 1 for the Consort diagram reflecting study recruitment and retention. Out of 176 people who completed the screening, 158 met eligibility requirements. We contacted 96 participants sequentially from this list until we reached a sample of 20 participants. Because three participants withdrew after consenting, 17 total participants received the Coping Crew intervention. There was no single reason why participants withdrew: one participant did not want to download the mobile app, another participant expressed concerns related to their visa, and another did not reside in Ohio.

Potential participants first completed a screening survey online using Qualtrics. Eligible and interested participants completed a baseline virtual appointment consisting of a structured diagnostic interview and a battery of self-report measures. Following the baseline appointment, participants received Coping Crew, which consisted of five intervention sessions over six weeks. Next, participants completed a battery of self-report measures at post-intervention and 1- and 3-month follow-up. At post-intervention, participants completed a semi-structured interview containing questions designed to capture perceived acceptability, usefulness, and effectiveness of the intervention, in addition to the user experience of the accompanying mobile app. Participants were compensated up to $130 for their participation.

### Development and structure of the Coping Crew intervention

Iteratively designed from focus groups and case studies, Coping Crew is a modular CBT intervention. We reviewed and adapted content from existing interventions for the anxiety sensitivity (AS) module (Computerized Anxiety Sensitivity Treatment [14,24,47]; and the intolerance of uncertainty (IU) module [38]. Principles from “A Therapist’s Guide to Brief Cognitive Behavioral Therapy [58]” served as a reference for developing modules and homework.

The intervention consisted of five weekly 60-minute group sessions delivered virtually via Microsoft Teams by two masters-level PhD graduate student clinicians (one male and one female) with CBT training. The first four sessions occurred weekly with a 2-week break between the fourth and fifth session to provide additional time for skills building. Groups ran one at a time from September 2021 to June 2022. Within each group, participants unable to attend a group session completed a make-up session with a clinician one-on-one before the next session.

### Content of the Coping Crew intervention

Coping Crew is a modular CBT intervention that addresses emotional distress through a series of five remote group sessions. The program uses the modular CBT approach by focusing on a different topic each week: AS, IU, and loneliness. In all sessions, homework from the preceding week is reviewed in session with allocated time to address participant questions or concerns. The first session introduces the program, establishes group norms, and promotes voluntary intra-group sharing to enhance corrective feedback. Clinicians explain the CBT approach, and participants practice relaxation exercises.

The next three sessions each target a transdiagnostic risk factor for emotional distress using the same virtual group format. The second session introduces the role of anxiety as the body’s alarm system, and how to challenge misperceptions about anxiety sensations. Behavioral experiments (e.g., rapid breathing to mimic shortness of breath, or running in place to mimic a rapidly beating heart) are assigned to reinforce that anxiety sensations are benign. Session three introduces IU, its role in causing and maintaining anxiety, and common misperceptions about uncertainty. Participants are urged to challenge their own beliefs about uncertainty. Finally, participants design behavioral experiments that encourage confronting uncertainty. For example, in the pilot study, participants chose to delegate a task to a co-worker or answer a question in class they did not know the answer to with complete certainty. In session four, the focus shifts to social isolation experienced due to the pandemic and any accompanying loneliness. Strategies based on Behavioral Activation (BA) are introduced to encourage positive social interactions and challenge beliefs that inhibit interpersonal connection. Homework can include choosing social activities such as making small talk with a stranger or reaching out to an old friend.

The final session wraps up the intervention by revisiting all topics covered. In this session, clinicians summarize the information covered, encourage self-reflection, review strategies to maintain treatment gains, and discuss goal setting and values. Participants are provided with resources on how to obtain mental health care who to contact if experiencing a mental health crisis. This modular CBT-based intervention is designed to promote resilience in the face of anxiety, accompanying anxiety sensations, and social isolation.

### Mobile intervention component

The mobile component of the intervention used a HIPAA-secured mobile app available from Metricwire (metricwire.com) named mEMA. Over a 12-hour window, a static survey was delivered and then three additional surveys were quasi-randomly delivered no closer than an hour and a half apart over the remaining time. Participants completed a set of self-report questionnaires in the morning and visual analog scales (VAS) across all assessments. Cognitive challenges were delivered if VAS ratings were ≥ 5 on a 10-point scale. These cognitive challenges focused on “key takeaways” from each week’s intervention topic collaboratively developed by members of each group beginning in week two. Each group cohort generated their own “key takeaways” that challenged maladaptive cognitions related to each session’s content. For example, AS-specific challenges included “anxiety is just a feeling” and “the anxiety I feel now will pass.” IU-specific challenges included “even if things go off plan, I can adjust for that” and “uncertainty doesn’t mean something bad is going to happen” and loneliness-specific challenges included “avoiding social interactions won’t improve feeling alone.” After week two, whenever a participant reported anxiety or stress ≥ 5, one of these key takeaways was delivered randomly from a list of takeaways generated in-session. AS and IU-related key takeaways were randomly selected from after week three with a 75% likelihood of drawing an IU-related key takeaway. After week four, AS and IU key takeaways were equally delivered whenever a participant reported elevated anxiety or stress, and loneliness key takeaways were delivered whenever a participant reported elevated depression. The prompts for each emotional distress and the corresponding behavioral experiments evolved with the progression of the intervention, giving participants an interactive, personalized, and comprehensive experience.

### Feasibility, acceptability, and utility measures

The study employs a custom set of evaluative measures to gauge the feasibility of the intervention and associated assessment battery, and acceptability and utility of the intervention and mobile app. **Feasibility** was determined by attendance rates for assessment and intervention sessions, and rates of survey completion via mobile app. **Acceptability** was measured by perceived **acceptability of the intervention** and **acceptability of the mobile app,** measured by participants’ attitudes toward the intervention’s impact on AS, IU, loneliness, anxiety, stress, and depression, as well as the anticipated sustainability of clinical improvements. Perceived **utility of the services provided** included questions focused on the ability of the intervention to reduce anxiety, stress, and loneliness and use of the mobile app to track weekly activities. All items were assessed on a 5-point Likert-like scale with higher scores indicating greater agreement with the item.

### Assessment measures

#### Anxiety Sensitivity Index-3.

The ASI-3 [59] is an 18-item measure that assesses fear of physical, cognitive, and observable anxiety sensations by measuring three AS subfactors (physical, cognitive, and social concerns). Items are assessed on a 5-point Likert-like scale with higher scores indicating greater AS. In the current study, reliability for the ASI-3 from baseline to follow-up was excellent (Cronbach’s alpha [α] =.91−.95).

#### Intolerance of Uncertainty Scale short form.

The IUS-12 is a 12-item measure that assesses the degree to which individuals can tolerate the uncertainty of ambiguous situations, cognitive and behavioral responses to uncertainty, perceived implications of uncertainty, and attempts to control the future through two factors: prospective anxiety and inhibitory anxiety [60]. Items are assessed on a 5-point Likert-like scale where 1 indicates that the respondent does not find the statement to be characteristic of themselves, and 5 indicates that the respondent finds the statement to be very characteristic of themselves. In the current study, reliability for the IUS-12 from baseline to 1-week follow-up was good to excellent (α = .83−.93).

#### NIH Toolbox: Adult Social Relationship Scales (Loneliness).

The National Institute of Health (NIH) Toolbox Loneliness Scale [61] is 5-item measure within the NIH Toolbox Adult Social Relationship Scales that assesses loneliness, conceptualized as the perception that one is lonely or socially isolated from others. Items are assessed on a 5-point scale with 1 indicating the respondent never has the experience, and 5 indicating that the respondent always has the experience. In the current study, reliability for the NIH Loneliness scale from baseline to 2-week follow-up was good to excellent (α = .87−.96).

#### PROMIS Negative Affect Scales: Anxiety & depression.

The Patient Reported Outcomes Measurement Information System (PROMIS) Negative Affect scales include measures for assessing anxiety and depression [62]. The PROMIS Anxiety item bank consists of 29 items, and the PROMIS Depression item bank consists of 28 items. We utilized the short forms of both measures, containing 8 items each. Reliability for the PROMIS Depression scale from baseline to 3-month follow-up was excellent (α = .95−.97).

#### COVID Impact Battery: COVID-19 Worry Scale.

The COVID Impact Battery (CIB) captures the influence of the COVID-19 pandemic on individual mental health across three COVID-19-related scales: behavior, worry, and disability [63]. The COVID-19 Worry scale is an 11-item measure that assesses COVID-related stress, associated with avoidance of infection via safety behaviors, self-checking, and reassurance-seeking (i.e., utilization of the healthcare system), as well as worry about socioeconomic impacts of the pandemic [64]. Items are assessed on 0–4 scales with higher scores capturing increased worry. In the current study, reliability for the CIB Worry scale from baseline to 3-month follow-up was good to excellent (α = .84−.92).

### EMA measures

During the intervention, participants completed four daily brief surveys. The first survey was sent at the beginning of a 12-hour period predetermined by the client and clinician, followed by three surveys delivered randomly throughout the remaining 12-hour window. All surveys were delivered at least 90 minutes apart. The purpose of the personalized time period was to maximize participant engagement, by increasing likelihood of survey completion via convenience. The brief surveys were designed to take approximately one minute to complete. EMA measures included the CIB and four Visual Analog Scales (VAS): Anxiety, Depression, Stress, and Loneliness. VAS measures were formatted as slider bars for participants to rate their levels on a 1–100 scale (e.g., How anxious are you now?) with higher scores indicating more severe levels of the rated construct.

### Data analytic plan

This study was a one-arm pilot acceptability and feasibility study, registered on clinicaltrials.gov (NCT#05019053). Reporting was done in accordance with the Consolidated Standards of Reporting Trials (CONSORT) guidelines, adopted for one-arm trials [65]. The main outcomes of the study, acceptability, feasibility, and utility ratings, were first calculated by examining mean item-level scores across several measures modified to capture participant ratings of acceptability, feasibility, and utility. Acceptability and feasibility were also assessed through monitoring recruitment, retention, and use of the mobile app.

Exploratory analyses, conducted as repeated measures ANOVA, examined changed in mean AS, IU, loneliness, anxiety, depression, and worry related to the COVID-19 pandemic from baseline to post-intervention and 1-month follow-up (see S1 Table). Mauchly’s test was used to determine if the assumption of sphericity was met. If it was not met, the Greenhouse Geisser statistic was used to determine the significance of the tests of within-subjects effects. Because this trial was not designed to be powered to find all but the largest of effect sizes, we were primarily interested in standardized effect size estimates (*d*w) which was calculated as the change in scores divided by the pooled baseline standard deviation.

## Results

### Eligibility criteria met for enrollment

Eligibility criteria included elevations of at least 1 *SD* above the mean on at least one risk factor: AS, IU, or loneliness. At baseline, 13 participants had elevated ASI-3 scores, 17 participants had elevated IUS-12 scores, and6 participants had elevated NIH Loneliness scores.

### Acceptability, feasibility, and utility

Acceptability, feasibility, and utility were examined across attendance and performance metrics as well as response to Likert-like questions. Out of 20 participants who consented and completed the baseline session, 3participants withdrew leaving 17 participants (85%) who received Coping Crew. All 17 participants attended one or more treatment sessions, and 15 attended all five intervention sessions. All 17 participants completed their baseline and 1-month follow-up survey batteries. Only one participant did not complete the 3-month follow-up battery. Regarding missing sessions, zero participants missed more than one session and lack of attendance varied by session number (session two = 2 participants; session three = 1 participant; session four = 1 participant). This suggests that the deliverability of the intervention and corresponding assessment protocol are feasible. Performance on the daily surveys via the mobile app was mixed. Only 6 (35%) of 17 participants completed 80% or more of the surveys delivered to their mobile app. However, 16 of 17 (94%) completed at least one survey daily 80% of the days. Participants completed an average of 13 (range = 2–23) behavioral experiments assigned as daily homework.

Participants rated perceived efficacy of the intervention component delivered by clinicians across 10 items on a scale of 1 = “strongly disagreed with the item” to 5 = “strongly agreed” (see Top Panel of Table 1). The average item rating was 4.23 (range = 3.75–4.63), reflecting that on average, participants agreed on acceptability of the intervention for anxiety, stress, and depression. Of note, participants strongly agreed (*M *= 4.63, *SD *= .62) that they would recommend this approach to others who are lonely and anxious/stressed.

**Table 1:** Ratings of acceptability.

Acceptability of the Intervention	Mean	SD
This was an acceptable intervention for loneliness	3.75	0.77
This was an acceptable intervention for anxiety	4.31	0.70
This intervention was useful	4.38	0.72
I benefitted from this intervention	4.19	0.66
This intervention helped me reduce my depression	3.88	0.72
This intervention helped me reduce my stress	4.06	0.68
I expect to maintain improvements	4.13	0.72
This intervention is a good approach for addressing anxiety/stress	4.38	0.62
This intervention could be helpful to others who are lonely and anxious/stressed	4.56	0.51
I would recommend this intervention to others who are lonely and anxious/stressed	4.63	0.62
**Acceptability of the Mobile Application**		
The number of times I had to complete the surveys on my mobile device was acceptable	3.69	1.01
The daily surveys were useful	3.88	0.89
The number of daily surveys I completed was just right	3.50	0.89
I benefitted from using my telephone to complete the homework	4.25	0.93
The mobile app helped me reduce my loneliness	2.69	0.70
The mobile app helped me reduce my anxiety	3.69	1.14
I found the prompts regarding anxiety, uncertainty, and loneliness to be helpful	4.06	0.85
The mobile app worked well	4.44	0.81
**Utility of the Services Provided**		
Having the focus of the treatment be on reducing anxiety, stress, and loneliness	4.25	.77
Addressing anxiety, stress, and loneliness through a cognitive-behavioral intervention.	4.31	.87
Being able to address anxiety, stress, and loneliness through telehealth appointments	3.81	1.05
Learning and practicing relaxation techniques	4.00	.97
Using your mobile phone for tracking your weekly activities	4.06	1.06
Having to complete weekly and sometimes daily homework	3.50	.97
Working with your therapist	4.06	.93

*Notes.* Items were scored from 1 = strongly disagree to 5 = strongly agree.

Participants rated perceived efficacy of the mobile app components of the intervention across 8 items using the same 1–5 Likert scale (see Middle Panel of Table 1). The average rating was 3.77 (range = 2.69–4.44), indicating participants found the mobile app acceptable in a variety of ways, including finding the daily surveys useful (*M *= 3.88, *SD *= .89), and using their cell phone to complete homework (*M *= 4.25, *SD *= .93). Participants strongly agreed (*M *= 4.44, *SD* = .81) that the mobile app worked well. Of note, the average level of agreement on whether the mobile app was helpful for loneliness was neutral, meaning participants neither agreed or disagreed that it was helpful.

### Missing data analysis

We examined differences in baseline mean scores between participants who were missing data versus participants who were not missing any data. A significant difference in PROMIS Depression scale scores was found, *t*(15) = −2.06, *p *= .03. Participants who provided data at all assessment periods had lower depression scores (*M *= 15.50, *SD* = 5.66; *n* = 8) than did participants who did not provide data at all assessment periods (*M *= 21.56, *SD* = 6.39; *n* = 9). No other differences were found.

### Baseline descriptive statistics and correlations

Table 2 contains correlations between baseline variables as well as sample means. ASI-3, IUS-12, and NIH Loneliness scores were significantly correlated (*r*s = .51−.72, *p*s < .05). All three risk factors were associated with the PROMIS Anxiety scale (*r*s = .55−.79, *p*s < .05). The NIH Loneliness scale was also associated with scores on the PROMIS Depression scale (*r *= .64, *p *< .05) and COVID-19 Worry scale (*r *= .50, *p *< .05).

**Table 2:** Means and bivariate correlations and between outcome measures at baseline.

Measure	1	2	3	4	5	6	7	8
1. ASI-3	–	.72^**^	.63^**^	.69^**^	.44	.42	−.09	−.11
2. IUS-12	–	–	.51^*^	.55^*^	.19	.17	−.09	.20
3. NIH Loneliness	–	–	–	.68^*^	.64^**^	.50^*^	.10	−.02
4. PROMIS Anxiety	–	–	–	–	.55^*^	.43	−.12	−.16
5. PROMIS Depression	–	–	–	–	–	.40	.18	−.07
6. CIB Worry	–	–	–	–	–	–	.16	−.57^*^
7. Age	–	–	–	–	–	–	–	.34
8. Sex	–	–	–	–	–	–	–	–
Mean	34.12	53.88	14.94	25.29	18.71	16.29	22.35	–
SD	12.14	9.85	5.37	6.89	6.64	7.85	4.46	–

*Notes.* ASI-3 = Anxiety Sensitivity Index-3; IUS-12 = Intolerance of Uncertainty Scale-12; NIH = National Institutes of Health; PROMIS = Patient-Reported Outcomes Measurement Information System. CIB = Coronavirus Information Battery.

* *p* = ≤.05.

** *p* = ≤.01.

### Exploratory analyses: Repeated measures ANOVAs

Exploratory analyses were conducted to assess changes in transdiagnostic risk factors and emotional distress symptoms from baseline to follow-up. Preliminary reductions in AS, IU, and loneliness were observed from baseline to follow-up. The results suggest medium-to-large effect size reductions, though they should be interpreted with caution due to the small sample size. Detailed results of the repeated measures ANOVA, including effect sizes, are presented in S1 Table.

## Discussion

This study was a preliminary feasibility, acceptability, and utility one armed pilot trial for Coping Crew, a modular group CBT-based transdiagnostic intervention. To encourage use of in-session skills developed, we designed a mobile app to supplement the treatment. The intervention and assessment battery were feasible, acceptable, and had utility in addressing the targeted transdiagnostic risk factors. Although the mobile app was rated as acceptable and had utility in aiding delivery of the intervention, rates of completion were lower than we had proposed as our threshold for acceptability.

We determined that Coping Crew was acceptable and feasible based on recruitment and retention rates as well as ratings on measures assessing agreement that aspects of the intervention were acceptable and useful. We retained 85% of participants (17 of 20) consented to receive the intervention, and 88% (15 of 17) of participants attended all five intervention sessions as well as the 3-month follow-up and 94% (16) attended the 1-month follow-up. On average, the intervention content was rated as acceptable. Importantly, on average, participants strongly agreed that they would recommend this intervention to others who are lonely and anxious/stressed. This suggests that client perceptions of Coping Crew align with the intended purpose of the treatment.

In contrast to the almost universally positive perceptions of the telehealth component, perceptions about the mobile app were more mixed. We hypothesized, a priori, that if 80% of the participants completed at least 80% of the surveys delivered to their phone, we would consider that as evidence that the mobile app was a feasible accompaniment to the intervention. Only six participants (35%) met this threshold. However, 94% of participants completed at least one survey on more than 80% of the days. Evidence for the acceptability and utility of the mobile app was mixed. On average, participants agreed they completed an acceptable number of surveys daily, that they benefitted from using their phone to complete their homework, and that the prompts they received regarding anxiety, uncertainty, and loneliness were helpful. We did learn from responses to open- and close-ended questions that one participant was opposed to use of the mobile app. This participant disagreed to strongly disagreed that the mobile app to track their mood was a useful adjunct to the treatment. It is possible there may be barriers to engaging with mobile apps for some participants. For example, older adults, low-income groups, and rural populations may not have the necessary resources or digital literacy skills to benefit from an app [66]. Others may be concerned about data privacy and confidentiality [67]. There is an emerging literature on engagement efforts for mHealth interventions; more is needed to understand which factors may limit research participants or clinical patient from benefitting from mobile app interventions and supplements and whether acceptable substitutes exist.

Detailed statistical results, including effect sizes, are available in the supplementary material to support transparency and future meta-analyses. Effect sizes obtained in one-armed pilot trials must be interpreted with caution due to the lack of a control condition. Further, our small sample size also precludes estimations of treatment effects. Preliminary analyses suggest that reductions in AS, IU, and loneliness were maintained at follow-up assessments, supporting the potential for targeting these transdiagnostic risk factors in future, more rigorous trials. However, no significant reductions were observed in depression symptoms, which may reflect limitations of the intervention or sample characteristics.

A further benefit of validating Coping Crew via a SMART trial is the ability to test the inherent modularity of the intervention. Modular treatments have emerged as a promising approach that bridges the gap between traditional psychotherapy practices, driven by clinician intuition, and manualized treatments, characterized by rigid protocols [68]. Whereas traditional approaches rely on clinicians’ adaptability to meet clients’ evolving needs, manualized treatments provide structure at the cost of flexibility. In contrast, modular treatments allow for manualized treatments to be utilized based solely on participants’ current needs [68]. Modular frameworks acknowledge the finite nature of therapeutic tools and offer the potential for “continuous scaling” from a common treatment framework [68]. For instance, in the development of Coping Crew, an intervention currently under development, principles derived from both first and third-wave cognitive-behavioral therapy (CBT) as well as mindfulness practices are utilized to explore the intricate interplay among thoughts, feelings, and behaviors, with a specific focus on the psychological modifiability of thoughts and behaviors [69].

### Limitations

Despite grounding our study in prior literature, several limitations should be acknowledged. First, reliance on self-report measures introduces potential biases associated with memory processes and response tendencies. Whereas real-time data collection via EMA may mitigate some biases, further exploration of alternative measurement approaches is warranted. Second, the small sample size and convenience sampling resulting in a primarily White non-Hispanic and female sample limit the generalizability of our findings. Further, the lack of a control group limits interpretation of change. Multi-arm fully powered RCT designs are needed to fully explore efficacy of Coping Crew as well as to identify mechanisms underlying treatment effects. These limitations provide directions for future research and further highlight the need for an RCT comparing Coping Crew to a well-validated control condition.

## Conclusion

This study served as a one-arm pilot feasibility, acceptability, and utility trial. Results were promising for the intervention and assessment protocol. Considerations for next steps include how to increase engagement in the mobile app and fitting intervention modules to sample characteristics. These findings leave us eager to conduct next stages in this modular transdiagnostic intervention that includes a mobile app component to supplement the intervention with limited clinical effort.

## Supporting information

S1 Table Detailed effect-size estimates for all outcomes. (DOCX)

S1 File CONSORT checklist pilot trial corrected. (DOCX)

S2 File IRB approved protocol. (PDF)

S3 File Trend statement TREND checklist. (PDF)

## References

[1] Dozois DJA. Anxiety and depression in Canada during the COVID-19 pandemic: A national survey. Canadian Psychol / Psychologie canadienne. 2021;62(1):136–42. doi: 10.1037/cap0000251

[2] Cordaro M, Grigsby TJ, Howard JT, Deason RG, Haskard-Zolnierek K, Howard K. Pandemic-specific factors related to generalized anxiety disorder during the initial COVID-19 protocols in the United States. Issues Ment Health Nurs. 2021;42(8):747–57. doi: 10.1080/01612840.2020.1867675 33480832

[3] Khoury JMB, Watt MC, MacLean K. Anxiety sensitivity mediates relations between mental distress symptoms and medical care utilization during COVID-19 pandemic. Int J Cogn Ther. 2021;14(3):515–36. doi: 10.1007/s41811-021-00113-x 34178209 PMC8216097

[4] Bueno-Notivol J, Gracia-García P, Olaya B, Lasheras I, López-Antón R, Santabárbara J. Prevalence of depression during the COVID-19 outbreak: A meta-analysis of community-based studies. Int J Clin Health Psychol. 2021;21(1):100196. doi: 10.1016/j.ijchp.2020.07.007 32904715 PMC7458054

[5] Moscona G, Moscona G, Gozansky E, Gozansky E, Okon-Singer H, Okon-Singer H. Identifying variables that predict depression following the general lockdown during the COVID-19 pandemic. Biol Psychiatry. 2021;89(9):S150. doi: 10.1016/j.biopsych.2021.02.386 PMC8165248 34079505

[6] Cromarty P, Gallagher D, Watson J. Remote delivery of CBT training, clinical supervision and services: in times of crisis or business as usual. Cogn Behav Therap. 2020;13:e33. doi: 10.1017/S1754470X20000343 34191942 PMC7468679

[7] Allan NP, Albanese BJ, Short NA, Raines AM, Schmidt NB. Support for the general and specific bifactor model factors of anxiety sensitivity. Person Individual Differ. 2015;74:78–83. doi: 10.1016/j.paid.2014.10.003

[8] Watson D, Levin-Aspenson HF, Waszczuk MA, Conway CC, Dalgleish T, Dretsch MN, et al. Validity and utility of Hierarchical Taxonomy of Psychopathology (HiTOP): III. Emotional dysfunction superspectrum. World Psychiatry. 2022;21(1):26–54. doi: 10.1002/wps.20943 35015357 PMC8751579

[9] Kessler RC, Chiu WT, Demler O, Merikangas KR, Walters EE. Prevalence, severity, and comorbidity of 12-month DSM-IV disorders in the National Comorbidity Survey Replication. Arch Gen Psychiatry. 2005;62(6):617–27. doi: 10.1001/archpsyc.62.6.617 15939839 PMC2847357

[10] Hayes SC, Hofmann SG. The third wave of cognitive behavioral therapy and the rise of process-based care. World Psychiatry. 2017;16(3):245–6. doi: 10.1002/wps.20442 28941087 PMC5608815

[11] Struijs SY, de Jong PJ, Jeronimus BF, van der Does W, Riese H, Spinhoven P. Psychological risk factors and the course of depression and anxiety disorders: A review of 15 years NESDA research. J Affect Disord. 2021;295:1347–59. doi: 10.1016/j.jad.2021.08.086 34706448

[12] Roberge P, Provencher MD, Gaboury I, Gosselin P, Vasiliadis H-M, Benoît A, et al. Group transdiagnostic cognitive-behavior therapy for anxiety disorders: a pragmatic randomized clinical trial. Psychol Med. 2020;52(13):2460–70. doi: 10.1017/s0033291720004316 PMC9647541 33261700

[13] Schaeuffele C, Bär J, Buengener I, Grafiadeli R, Heuthaler E, Strieder J, et al. Transdiagnostic processes as mediators of change in an internet-delivered intervention based on the unified protocol. Cogn Ther Res. 2021;46(2):273–86. doi: 10.1007/s10608-021-10272-y

[14] Schmidt NB, Capron DW, Raines AM, Allan NP. Randomized clinical trial evaluating the efficacy of a brief intervention targeting anxiety sensitivity cognitive concerns. J Consult Clin Psychol. 2014;82(6):1023–33. doi: 10.1037/a0036651 24821096

[15] Schmidt NB, Raines AM, Allan NP, Zvolensky MJ. Anxiety sensitivity risk reduction in smokers: A randomized control trial examining effects on panic. Behav Res Ther. 2016;77:138–46. doi: 10.1016/j.brat.2015.12.011 26752327 PMC4752863

[16] Reiss S, Peterson RA, Gursky DM, McNally RJ. Anxiety sensitivity, anxiety frequency and the prediction of fearfulness. Behav Res Ther. 1986;24(1):1–8. doi: 10.1016/0005-7967(86)90143-9 3947307

[17] Taylor S, Cox BJ. An expanded anxiety sensitivity index: evidence for a hierarchic structure in a clinical sample. J Anxiety Disord. 1998;12(5):463–83. doi: 10.1016/s0887-6185(98)00028-0 9801964

[18] Allan NP, Norr AM, Boffa JW, Durmaz D, Raines AM, Schmidt NB. Examining the unique relations between anxiety sensitivity factors and suicidal ideation and past suicide attempts. Psychiatry Res. 2015;228(3):441–7. doi: 10.1016/j.psychres.2015.05.066 26154817

[19] Lejuez CW, Paulson A, Daughters SB, Bornovalova MA, Zvolensky MJ. The association between heroin use and anxiety sensitivity among inner-city individuals in residential drug use treatment. Behav Res Therapy. 2006;44(5):667–77. doi: 10.1016/j.brat.2005.04.006 16002042

[20] Olatunji BO, Wolitzky-Taylor KB. Anxiety sensitivity and the anxiety disorders: a meta-analytic review and synthesis. Psychol Bull. 2009;135(6):974–99. doi: 10.1037/a0017428 19883144

[21] Stanley IH, Boffa JW, Rogers ML, Hom MA, Albanese BJ, Chu C, et al. Anxiety sensitivity and suicidal ideation/suicide risk: A meta-analysis. J Consult Clin Psychol. 2018;86(11):946–60. doi: 10.1037/ccp0000342 30335426 PMC6469498

[22] Rogers AH, Bogiaizian D, Salazar PL, Solari A, Garey L, Fogle BM, et al. COVID-19 and anxiety sensitivity across two studies in Argentina: Associations with COVID-19 worry, symptom severity, anxiety, and functional impairment. Cognit Ther Res. 2021;45(4):697–707. doi: 10.1007/s10608-020-10194-1 33424059 PMC7778696

[23] Çelik E, Biçener E, Bayın Ü, Uğur E. Mediation role of anxiety sensitivity on the relationships between intolerance of uncertainty and fear of COVID-19. An Psicol. 2022;38(1):1–6. doi: 10.6018/analesps.469511

[24] Schmidt NB, Norr AM, Allan NP, Raines AM, Capron DW. A randomized clinical trial targeting anxiety sensitivity for patients with suicidal ideation. J Consult Clin Psychol. 2017;85(6):596–610. doi: 10.1037/ccp0000195 28287798

[25] Short NA, Allan NP, Raines AM, Schmidt NB. The effects of an anxiety sensitivity intervention on insomnia symptoms. Sleep Med. 2015;16(1):152–9. doi: 10.1016/j.sleep.2014.11.004 25547037

[26] Short NA, Fuller K, Norr AM, Schmidt NB. Acceptability of a brief computerized intervention targeting anxiety sensitivity. Cogn Behav Ther. 2017;46(3):250–64. doi: 10.1080/16506073.2016.1232748 27712458

[27] Carleton RN, Mulvogue MK, Thibodeau MA, McCabe RE, Antony MM, Asmundson GJG. Increasingly certain about uncertainty: Intolerance of uncertainty across anxiety and depression. J Anxiety Disord. 2012;26(3):468–79. doi: 10.1016/j.janxdis.2012.01.011 22366534

[28] Hale W, Richmond M, Bennett J, Berzins T, Fields A, Weber D, et al. Resolving uncertainty about the intolerance of uncertainty scale-12: application of modern psychometric strategies. J Pers Assess. 2016;98(2):200–8. doi: 10.1080/00223891.2015.1070355 26542301 PMC4809643

[29] Huntley CD, Young B, Tudur Smith C, Fisher PL. Uncertainty and test anxiety: Psychometric properties of the Intolerance of Uncertainty Scale – 12 (IUS-12) among university students. Int J Educ Res. 2020;104:101672. doi: 10.1016/j.ijer.2020.101672

[30] Ren L, Wei Z, Li Y, Cui L-B, Wang Y, Wu L, et al. The relations between different components of intolerance of uncertainty and symptoms of generalized anxiety disorder: a network analysis. BMC Psychiatry. 2021;21(1):448. doi: 10.1186/s12888-021-03455-0 34507563 PMC8431915

[31] Lind C, Boschen MJ. Intolerance of uncertainty mediates the relationship between responsibility beliefs and compulsive checking. J Anxiety Disord. 2009;23(8):1047–52. doi: 10.1016/j.janxdis.2009.07.005 19656653

[32] Shapiro MO, Short NA, Morabito D, Schmidt NB. Prospective associations between intolerance of uncertainty and psychopathology. Person Individual Differ. 2020;166:110210. doi: 10.1016/j.paid.2020.110210

[33] Boswell JF, Thompson-Hollands J, Farchione TJ, Barlow DH. Intolerance of uncertainty: a common factor in the treatment of emotional disorders. J Clin Psychol. 2013;69(6):630–45. doi: 10.1002/jclp.21965 23381685 PMC3712497

[34] Hamama-Raz Y, Goodwin R, Leshem E, Ben-Ezra M. Intolerance of uncertainty and mental health during the COVID-19 pandemic: The role of anger as a moderator. J Psychiatr Res. 2021;138:50–2. doi: 10.1016/j.jpsychires.2021.03.032 33819877 PMC9750187

[35] Voitsidis P, Nikopoulou VA, Holeva V, Parlapani E, Sereslis K, Tsipropoulou V, et al. The mediating role of fear of COVID-19 in the relationship between intolerance of uncertainty and depression. Psychol Psychother. 2021;94(3):884–93. doi: 10.1111/papt.12315 33216444 PMC7753422

[36] Dai W, Meng G, Zheng Y, Li Q, Dai B, Liu X. The impact of intolerance of uncertainty on negative emotions in COVID-19: Mediation by pandemic-focused time and moderation by perceived efficacy. Int J Environ Res Public Health. 2021;18(8):4189. doi: 10.3390/ijerph18084189 33920976 PMC8103505

[37] Allan NP, Volarov M, Koscinski B, Pizzonia KL, Potter K, Accorso C, et al. Lonely, anxious, and uncertain: Critical risk factors for suicidal desire during the COVID-19 pandemic. Psychiatry Res. 2021;304:114144. doi: 10.1016/j.psychres.2021.114144 34364010 PMC8442981

[38] Shapiro MO, Allan NP, Raines AM, Schmidt NB. A randomized control trial examining the initial efficacy of an intolerance of uncertainty focused psychoeducation intervention. J Psychopathol Behav Assess. 2022;45(2):379–90. doi: 10.1007/s10862-022-10002-y

[39] Dugas MJ, Sexton KA, Hebert EA, Bouchard S, Gouin J-P, Shafran R. Behavioral experiments for intolerance of uncertainty: a randomized clinical trial for adults with generalized anxiety disorder. Behav Ther. 2022;53(6):1147–60. doi: 10.1016/j.beth.2022.05.003 36229113

[40] van der Heiden C, Muris P, van der Molen HT. Randomized controlled trial on the effectiveness of metacognitive therapy and intolerance-of-uncertainty therapy for generalized anxiety disorder. Behav Res Ther. 2012;50(2):100–9. doi: 10.1016/j.brat.2011.12.005 22222208

[41] Calati R, Ferrari C, Brittner M, Oasi O, Olié E, Carvalho AF, et al. Suicidal thoughts and behaviors and social isolation: A narrative review of the literature. J Affect Disord. 2019;245:653–67. doi: 10.1016/j.jad.2018.11.022 30445391

[42] Hawkley LC, Cacioppo JT. Loneliness matters: a theoretical and empirical review of consequences and mechanisms. Ann Behav Med. 2010;40(2):218–27. doi: 10.1007/s12160-010-9210-8 20652462 PMC3874845

[43] Solmi M, Veronese N, Galvano D, Favaro A, Ostinelli EG, Noventa V, et al. Factors associated with loneliness: an umbrella review of observational studies. J Affect Disord. 2020;271:131–8. doi: 10.1016/j.jad.2020.03.075 32479308

[44] Allen RL, Oshagan H. The UCLA loneliness scale: Invariance of social structural characteristics. Person Individual Differ. 1995;19(2):185–95. doi: 10.1016/0191-8869(95)00025-2

[45] McClelland H, Evans JJ, Nowland R, Ferguson E, O’Connor RC. Loneliness as a predictor of suicidal ideation and behaviour: a systematic review and meta-analysis of prospective studies. J Affect Disord. 2020;274:880–96. doi: 10.1016/j.jad.2020.05.004 32664029

[46] Allan NP, Boffa JW, Raines AM, Schmidt NB. Intervention related reductions in perceived burdensomeness mediates incidence of suicidal thoughts. J Affect Disord. 2018;234:282–8. doi: 10.1016/j.jad.2018.02.084 29554617 PMC6434690

[47] Schmidt NB, Capron D, Raines AM, Albanese B, Short N, Mathes BM, et al. Evaluating the long-term (Three Year) durability of brief interventions targeting risk factors for psychopathology. J Anxiety Disord. 2023;96:102710. doi: 10.1016/j.janxdis.2023.102710 37058765 PMC13229105

[48] Käll A, Bäck M, Welin C, Åman H, Bjerkander R, Wänman M, et al. Therapist-guided internet-based treatments for loneliness: a randomized controlled three-arm trial comparing cognitive behavioral therapy and interpersonal psychotherapy. Psychother Psychosom. 2021;90(5):351–8. doi: 10.1159/000516989 34182552

[49] Schaeuffele C, Schulz A, Knaevelsrud C, Renneberg B, Boettcher J. CBT at the crossroads: the rise of transdiagnostic treatments. J Cogn Ther. 2020;14(1):86–113. doi: 10.1007/s41811-020-00095-2

[50] Ellard KK, Bentley KH, Maimone JS, Uribe S. Transdiagnostic CBT for anxiety and depressive disorders. In: Sprich SE, Petersen T, Wilhelm S, editors. The Massachusetts General Hospital Handbook of Cognitive Behavioral Therapy. Cham: Springer International Publishing; 2023. p. 343–58. doi: 10.1007/978-3-031-29368-9_23

[51] McHugh RK, Murray HW, Barlow DH. Balancing fidelity and adaptation in the dissemination of empirically-supported treatments: The promise of transdiagnostic interventions. Behav Res Ther. 2009;47(11):946–53. doi: 10.1016/j.brat.2009.07.005 19643395 PMC2784019

[52] Kazantzis N, Miller AR. A comprehensive model of homework in cognitive behavior therapy. Cogn Ther Res. 2021;46(1):247–57. doi: 10.1007/s10608-021-10247-z

[53] Mausbach BT, Moore R, Roesch S, Cardenas V, Patterson TL. The relationship between homework compliance and therapy outcomes: an updated meta-analysis. Cognit Ther Res. 2010;34(5):429–38. doi: 10.1007/s10608-010-9297-z 20930925 PMC2939342

[54] McGinn LK, Sanderson WC. What allows cognitive behavioral therapy to be brief: Overview, efficacy, and crucial factors facilitating brief treatment. Clin Psychol. 2001.

[55] Heron KE, Everhart RS, McHale SM, Smyth JM. Using mobile-technology-based ecological momentary assessment (EMA) methods with youth: a systematic review and recommendations. J Pediatr Psychol. 2017;42(10):1087–107. doi: 10.1093/jpepsy/jsx078 28475765

[56] Shiffman S, Stone AA, Hufford MR. Ecological momentary assessment. Annu Rev Clin Psychol. 2008;4:1–32. doi: 10.1146/annurev.clinpsy.3.022806.091415 18509902

[57] Saulnier KG, Koscinski B, Flynt S, Accorso C, Allan NP. Brief observable anxiety sensitivity treatment: intervention development and a pilot randomized-controlled acceptability and feasibility trial to evaluate a brief intervention for anxiety sensitivity social concerns. Cogn Behav Ther. 2024;53(2):190–206. doi: 10.1080/16506073.2023.2288551 38014462

[58] Cully JA, Teten AL. A therapist’s guide to brief cognitive behavioral therapy. Houst Dep Veterans Aff South Cent MIRECC. 2008.

[59] Taylor S, Zvolensky MJ, Cox BJ, Deacon B, Heimberg RG, Ledley DR, et al. Robust dimensions of anxiety sensitivity: development and initial validation of the Anxiety Sensitivity Index-3. Psychol Assess. 2007;19(2):176–88. doi: 10.1037/1040-3590.19.2.176 17563199

[60] Carleton RN, Norton MAPJ, Asmundson GJG. Fearing the unknown: a short version of the Intolerance of Uncertainty Scale. J Anxiety Disord. 2007;21(1):105–17. doi: 10.1016/j.janxdis.2006.03.014 16647833

[61] Cyranowski JM, Zill N, Bode R, Butt Z, Kelly MAR, Pilkonis PA, et al. Assessing social support, companionship, and distress: National Institute of Health (NIH) Toolbox Adult Social Relationship Scales. Health Psychol. 2013;32(3):293–301. doi: 10.1037/a0028586 23437856 PMC3759525

[62] Pilkonis PA, Choi SW, Reise SP, Stover AM, Riley WT, Cella D, et al. Item banks for measuring emotional distress from the Patient-Reported Outcomes Measurement Information System (PROMIS®): depression, anxiety, and anger. Assessment. 2011;18(3):263–83. doi: 10.1177/1073191111411667 21697139 PMC3153635

[63] Schmidt NB, Allan NP, Koscinski B, Mathes BM, Eackles K, Accorso C, et al. COVID-19 impact battery: development and validation. J Psychopathol Behav Assess. 2022;44(2):326–43. doi: 10.1007/s10862-021-09919-7 34518734 PMC8427558

[64] Taylor S, Landry CA, Paluszek MM, Rachor GS, Asmundson GJG. Worry, avoidance, and coping during the COVID-19 pandemic: A comprehensive network analysis. J Anxiety Disord. 2020;76:102327. doi: 10.1016/j.janxdis.2020.102327 33137601 PMC7585364

[65] Eldridge SM, Chan CL, Campbell MJ, Bond CM, Hopewell S, Thabane L, et al. CONSORT 2010 statement: extension to randomised pilot and feasibility trials. BMJ. 2016;355:i5239. doi: 10.1136/bmj.i5239 27777223 PMC5076380

[66] Choi NG, DiNitto DM, Marti CN, Choi BY. Telehealth use among older adults during COVID-19: associations with sociodemographic and health characteristics, technology device ownership, and technology learning. J Appl Gerontol. 2022;41(3):600–9. doi: 10.1177/07334648211047347 34608821 PMC8847316

[67] Robillard JM, Wu JM, Feng TL, Tam MT. Prioritizing benefits: a content analysis of the ethics in dementia technology policies. J Alzheimers Dis. 2019;69(4):897–904. doi: 10.3233/JAD-180938 31104020

[68] Chorpita BF, Daleiden EL, Weisz JR. Modularity in the design and application of therapeutic interventions. Appl Prevent Psychol. 2005;11(3):141–56. doi: 10.1016/j.appsy.2005.05.002

[69] Hofmann SG, Asnaani A, Vonk IJJ, Sawyer AT, Fang A. The efficacy of cognitive behavioral therapy: a review of meta-analyses. Cognit Ther Res. 2012;36(5):427–40. doi: 10.1007/s10608-012-9476-1 23459093 PMC3584580

