# Supplementary material for: A one-arm pilot trial of a telehealth CBT-based group intervention targeting transdiagnostic risk for emotional distress
Source: PLoS One. 2025 Jun 18;20(6):e0303131. doi: 10.1371/journal.pone.0303131 (PMC12176177; doi:10.1371/journal.pone.0303131)
Supplement: S1 Table — (DOCX) [file pone.0303131.s001.docx]

**Supplementary Table 1. *Repeated measures ANOVA for study outcomes.***

|  | Baseline (1) | | Post-Intervention (2) | | Month 1 (3) | | Month 3 (4) | | Overall *F* | *d*^a^ |
| --- | --- | --- | --- | --- | --- | --- | --- | --- | --- | --- |
| **Variables** | **M** | **SD** | **M** | **SD** | **M** | **SD** | **M** | **SD** |  |  |
| ASI-3 | 34.12^2,4^ | 14.14 | 17.38^1^ | 14.15 | 21.53 | 14.16 | 20.06^1^ | 13.91 | 9.95* | 1.12 |
| IUS-12 | 53.88^2,3,4^ | 9.85 | 30.69^1^ | 9.51 | 31.41^1^ | 9.47 | 53.88^1^ | 9.85 | 51.06* | .87 |
| NIH Loneliness | 14.94 | 5.37 | 11.00 | 4.55 | 11.81 | 4.40 | 12.71 | 5.18 | 3.53* | .62 |
| PROMIS Anxiety | 25.29 | 6.89 | 21.56 | 7.13 | 21.59 | 6.53 | 23.00 | 6.39 | 1.43 | .54 |
| PROMIS Depression | 18.71 | 6.64 | 15.88 | 6.23 | 18.53 | 7.05 | 18.69 | 9.28 | .38 | .43 |
| CIB Worry | 16.29 | 7.85 | 12.56 | 9.19 | 13.35 | 8.92 | 13.12 | 9.77 | 1.45 | .48 |

*Notes.* ASI-3 = Anxiety sensitivity index-3; IUS-12 = Intolerance of uncertainty-12; CIB = Covid Impact Battery. ^1-4^Reflect group differences when comparing each group to the other groups. ^a^Cohen’s *d*, calculated as the change from baseline to post-intervention divided by the baseline standard deviation is provided from baseline to post-intervention scores.

*^*^p* < .05.
