# Supplementary material for: A one-arm pilot trial of a telehealth CBT-based group intervention targeting transdiagnostic risk for emotional distress
Source: PLoS One. 2025 Jun 18;20(6):e0303131. doi: 10.1371/journal.pone.0303131 (PMC12176177; doi:10.1371/journal.pone.0303131)
Supplement: S1 File — (DOCX) [file pone.0303131.s002.docx]

CONSORT 2010 Checklist for Pilot/Feasibility Trials (Corrected Version)

This checklist was completed based on the manuscript titled: 'A One-Arm Pilot Trial of a Telehealth CBT-Based Group Intervention Targeting Transdiagnostic Risk for Emotional Distress'.

| Section/Topic | Item No | Checklist Item | Reported on Page No |
| --- | --- | --- | --- |
| Title and abstract | 1a | Identification as a pilot or feasibility randomized trial in the title | Title Page |
| Title and abstract | 1b | Structured summary of pilot trial design, methods, results, and conclusions | Abstract, pp. 2–3 |
| Introduction | 2a | Scientific background and rationale for future definitive trial | Introduction, pp. 4–6 |
| Introduction | 2b | Specific objectives or research questions for pilot trial | Introduction, p. 7 |
| Methods: Trial design | 3a | Description of pilot trial design including allocation ratio | Methods, p. 8 |
| Methods: Trial design | 3b | Important changes to methods after pilot trial commencement | Not applicable |
| Methods: Participants | 4a | Eligibility criteria for participants | Methods, pp. 8–9 |
| Methods: Participants | 4b | Settings and locations where the data were collected | Methods, p. 8 |
| Methods: Participants | 4c | How participants were identified and consented | Methods, pp. 8–9 |
| Methods: Interventions | 5 | Interventions intended for each group | Methods, pp. 9–12 |
| Methods: Outcomes | 6a | Completely defined prespecified assessments or measurements | Methods, pp. 12–16 |
| Methods: Outcomes | 6b | Any changes to pilot trial outcomes after commencement | Not applicable |
| Methods: Outcomes | 6c | Prespecified criteria used to judge progression to full trial | Not applicable |
| Methods: Sample size | 7a | Rationale for numbers in the pilot trial | Methods, p. 8 |
| Methods: Sample size | 7b | Explanation of any interim analyses and stopping guidelines | Not applicable |
| Methods: Randomization | 8a | Method used to generate the random allocation sequence | Not applicable |
| Methods: Randomization | 8b | Type of randomization; details of restriction | Not applicable |
| Methods: Randomization | 9 | Allocation concealment mechanism | Not applicable |
| Methods: Randomization | 10 | Implementation: who generated and assigned participants | Not applicable |
| Methods: Blinding | 11a | Blinding after assignment to interventions | Not applicable |
| Methods: Blinding | 11b | Similarity of interventions | Not applicable |
| Methods: Statistical methods | 12 | Statistical methods for outcomes | Methods, pp. 16–17 |
| Results: Participant flow | 13a | Numbers of participants approached, eligible, assigned | Results, p. 17; Figure 1 |
| Results: Participant flow | 13b | Losses and exclusions after enrollment | Results, p. 17 |
| Results: Recruitment | 14a | Dates defining recruitment and follow-up periods | Methods, p. 11 |
| Results: Recruitment | 14b | Why the pilot trial ended or was stopped | Completed as planned |
| Results: Baseline data | 15 | Baseline demographic and clinical characteristics | Results, pp. 17–18; Table 2 |
| Results: Numbers analyzed | 16 | Number of participants included in each analysis | Results, p. 17 |
| Results: Outcomes and estimation | 17 | Results for each objective | Results, pp. 17–21 |
| Results: Ancillary analyses | 18 | Other analyses performed | Results, pp. 21–22 |
| Results: Harms | 19 | Important harms or unintended effects | Not reported; presumed none |
| Results: Harms | 19a | Other important unintended consequences | Not applicable |
| Discussion | 20 | Pilot trial limitations | Discussion, pp. 22–24 |
| Discussion | 21 | Generalisability of pilot trial methods and findings | Discussion, pp. 22–23 |
| Discussion | 22 | Interpretation consistent with objectives and findings | Discussion, pp. 22–24 |
| Discussion | 22a | Implications for progression from pilot to future definitive trial | Discussion, pp. 22–24 |
| Other information: Registration | 23 | Registration number and name of trial registry | Methods, p. 8 |
| Other information: Protocol | 24 | Where trial protocol can be accessed | Available upon request |
| Other information: Funding | 25 | Sources of funding and other support | Title page; Methods, p. 8 |
| Other information: Ethical approval | 26 | Ethical approval or approval by research review committee | Methods, p. 8 |
