# Supplementary material for: A one-arm pilot trial of a telehealth CBT-based group intervention targeting transdiagnostic risk for emotional distress
Source: PLoS One. 2025 Jun 18;20(6):e0303131. doi: 10.1371/journal.pone.0303131 (PMC12176177; doi:10.1371/journal.pone.0303131)
Supplement: S2 File — (PDF) [file pone.0303131.s003.pdf]

Protocol Information	
**Review Level:**	FULL
**Protocol Status:**	APPROVED
**Protocol Number:**	21-F-7
**Expiration Date:**	03/11/2022
**Approved By:**	cale
**Approved Date:**	03/24/2021 1:42:57 PM
**Form Type:**	ORIGINAL
**Form Status:**	APPROVED
**Waiver:**	A waiver of signature is granted on the screening consent form.

Protocol History	
**Form Type**	**Form Status**
ORIGINAL - 03/24/2021	APPROVED
ORIGINAL - 03/11/2021	REVISION REQUESTED
ORIGINAL - 02/15/2021	REVISION REQUESTED
ORIGINAL - 02/02/2021	REVISION REQUESTED

Toggle Comments | View Changes

People & Roles

Project Title: Pilot Testing a Virtual Group Intervention for COVID-19 Distress

College: College of Arts and Sciences

Name	Role	CI	CITI Training
Accorso, Catherine	PI	Yes	• Expires: 08/06/2022
Suhr, Julie	ADVISOR	No	• Expires: 06/02/2022
Allan, Nicholas	ASSISTANT		• Expires: 07/26/2021
Austin, Megan	ASSISTANT		• Expires: 09/03/2022
Koscinski, Brandon	ASSISTANT		• Expires: 08/06/2022
Burton, Christa	ASSISTANT		• Expires: 10/08/2022
Gooch, Caroline	ASSISTANT		• Expires: 09/10/2023
Sanchez, Carmen	ASSISTANT		• Expires: 01/27/2022
Pucci, Gabriella	ASSISTANT		• Expires: 08/11/2022
Pizzonia, Kendra	ASSISTANT		• Expires: 07/31/2022
David, Kevin	ASSISTANT		• Expires: 08/10/2022
Kolnogorova, Kateryna	ASSISTANT		• Expires: 09/10/2023
Potter, Kaley	ASSISTANT		• Expires: 01/15/2022
Saulnier, Kevin	ASSISTANT		• Expires: 08/06/2022
Chapman, Mychaela	ASSISTANT		• Expires: 08/07/2022
Elliott, Riley	ASSISTANT		• Expires: 08/18/2021

Moradi, Shahrzad	ASSISTANT	• Expires: 09/14/2023
Whittington, Taylor	ASSISTANT	• Expires: 01/26/2022
Williams, Taylor	ASSISTANT	• Expires: 10/20/2022

Funding Status, Study Timeline and Health & Safety

**Study Timeline**	
**Date you wish to begin**	03/29/2021
**Duration of study**	1 Year(s) 0 Month(s)

YES	NO	
✔		**Are you receiving support or applying for funding?**<br>**From who or what entity will you be receiving funding?**<br>Ohio University - Baker Fund Awards Committee<br>**Describe any consulting or other relationships anyone on the research team may have with this sponsor.**<br>N/A<br>**Funding will be used for:**<br>• Paying Participants<br>• Research Expenses (postage, equipment, travel, etc.)
	✔	**Does your protocol require work with human blood, human tissues, cell cultures derived from human cell lines, or virus/bacteria that is classified as bio risk II or above by the CDC? Ohio University EHS website**
	✔	**Does this project involve activities covered by the Health Insurance Portability and Accountability Act (HIPAA)?**

Review Level

REVIEW LEVEL: FULL

Yes	No	
	✔	**The probability and magnitude of harm or discomfort anticipated in the research are not greater in and of themselves than those ordinarily encountered in daily life or during the performance of routine physical or psychological examinations or tests (45 CFR 46.102(j)).**

Recruitment/Selection of Subjects

Maximum number of participants to be enrolled? If screening occurs, include the number of subjects that will need to be screened in order to get the number necessary for statistical significance. Please note that once the protocol is approved this number must not be exceeded without prior approval of an amendment.

20000

Characteristics of subjects

Adults
University Students

Criteria for selection of subjects (inclusion/exclusion).

There are two aims for which participants will be recruited. The first aim involves forming a panel of community stakeholders to inform the development of a brief intervention for distress. The second aim involves testing the acceptability, feasibility, and usability of this intervention across four groups.

FOR AIM 1:
Participants will be those affiliated with community partners that serve clients who may benefit from this, or similar, interventions. This community partner stakeholder advisory group (SAG) will include 4 members recruited from the community. These board members will agree to attend three meetings over the one year of the project. Our community partners will include one board member from each organization or entity through which we will recruit participants. Three community partners signed letters of support to be involved in this grant, namely the Athens City-County Health Department, the Online Education in Psychology Program at Ohio University, and Dr. Laurie Fox Psychological Services, LLC (see letters of support). We expect to recruit one more community partner in the upcoming weeks.

FOR AIM 2:
This intervention will be offered to university and community adults and current adult patients at the Ohio University Psychology and Social Work Clinic (PSWC).
Included participants will be those who:
a) express interest in participating in an intervention for COVID-19 related stress,
b) report above-average anxiety sensitivity (i.e., a score greater than 1 standard deviation above the mean on the Anxiety Sensitivity Index-3; Taylor et al., 2007), intolerance of uncertainty (i.e., a score greater than 1 standard deviation above the mean on the Intolerance of Uncertainty Scale - Short Form; Carleton et al., 2007), or loneliness (i.e., a score greater than 1 standard deviation above the mean on the NIH Toolbox Loneliness Scale; Cyranowski et al., 2013) and
c) own a smartphone and have internet access. Recent PEW polling indicates that 81% of participants own a smartphone, including 96% of those 18-29 and 92% of those 30-49.

Interested participants will contact the Factors of Emotional/Affective Risk Lab. Once participants express interest, they will be screened to ensure that they own a smartphone, have internet access, and report either above-average anxiety sensitivity, intolerance of uncertainty, or loneliness, making them eligible for this intervention. Participants who meet criteria from this initial screening will complete an enrollment process through the PSWC, including the informed consent document for this study.

Participants who have previously received other FEAR Lab interventions or participated in a focus group related to that intervention (20-X-184; 20-X-204) will be excluded due to the overlap of intervention material.

Participants excluded from the study will be provided with mental health resources (see attached) should they want more information or further treatment.

- ACCHD letter of support 9.20.20.pdf
- Psychology Community Engagement Letter Of Support -Hoyt.pdf
- OUPSWC Anxiety Group Stakeholder Letter Fox.pdf
- Resources (2) (1).docx

Description of how they will identify and recruit prospective participants.

FOR AIM 1:
Members of the SAG will be recruited with the assistance of the clinic director, Dr. Megan Austin. Potential community partners have been previously identified and will be contacted directly with information about the study and the responsibilities and benefits of SAG participation.

FOR AIM 2:
Because we intend to recruit participants through multiple methods, including an email with a link to the screening form that reaches a large percentage of OU faculty, staff, and students, we have estimated our maximum participants for the screening estimate.

Multiple methods will be used for the recruitment of participants for this study, including:
1. offering the intervention to current adult patients at the Ohio University PSWC. Current adult patients will be recruited for the intervention through word of mouth (i.e., hearing about the study from their current clinicians), phone calls to provide a list of services and flyers posted physically in the PSWC (see COPING CREW ad);
2. the use of social media (i.e., Facebook, Craigslist, Twitter), emails sent via the Ohio Information Technology Services to OU-affiliated faculty and staff, and flyers posted at local businesses to recruit community adults who are not current patients at the PSWC, and
3. the distribution of ads to our community partners (SAG members) to recruit community adults.

Interested prospective participants will complete an online screening survey or call the Factors of Emotional/Affective Risk Lab to complete the screening over the phone. For participants who complete the screening over the phone, the research assistant conducting the screening will use a computer to enter the participants' responses into the same online survey that participants who completed the online screening filled out. Screening survey responses will be downloaded on a weekly basis. Once the screening survey responses are downloaded, all responses on the online survey will be deleted. After participants are scheduled for the first study session or deemed ineligible for participation any identifying information will be deleted from

the screening survey dataset. Participants not meeting eligibility criteria for this study will be provided with a resource sheet for local mental health treatment resources (see attached document under project description).

All participants will complete an informed consent document for participation in the screening portion of this study. Eligible participants will also complete an informed consent document for participation in the intervention.

The goal is to recruit 24 participants across four groups for this intervention. Those not eligible for participation in this intervention will be informed of other virtual interventions we are offering in the clinic at affordable rates.

YES	NO	
	✔	**Are they accessing existing records for this study?**

Description of relationship and/or anyone on the research team's relationship with potential participants.

As members of OU and the broader community are recruited, it is possible that potential participants could know the PI or research assistants. All members of the research team have undergone training in the importance of confidentiality to minimize the risk of harm to participants in situations such as this.

Recruitment tools

- COPING CREW Facebook Ad 02.28.2021.docx
- COPING CREW online advertisement 02.28.2021.docx
- COPING CREW recruitment email 2.28.21.docx
- COPING CREW recruitment flyer 02.28.2021 (1).docx

Performance Sites/Location of Research

Using campus facilities

Project Description

Summary of this project

The coronavirus (COVID-19) pandemic is a significant psychological stressor that threatens the onset of a mental health crisis in the US. Fear and anxiety about COVID-19 and its economic impact, as well as loneliness due to the required social isolation, are driving the mental health impacts of COVID-19; in a recent Kaiser Family Foundation poll, 45% of respondents reported that the coronavirus has had a negative impact on their mental health (Panchal et al., 2020). This is reflected in Southeastern Ohio. In data we collected from 317 Ohio University faculty, staff, and students from late May to early June, 39% reported moderate-to-severe levels of anxiety, 41% reported moderate-to-severe levels of depression and 57% reported the COVID-19 outbreak was impacting their sense of social connection much or very much. Despite the significant community need for accessible and affordable care, there are currently no evidence-based interventions for individuals coping poorly with coronavirus-related distress. We have developed a virtual group-based intervention targeting cognitive biases that amplify the experience of stress and anxiety (i.e., amplifying cognitions; Coping with Coronavirus-Related Emotion and Worry [COPING CREW]). The next step in developing this intervention in a scientifically rigorous manner is to refine the manual and procedures and conduct a pilot test of the intervention.

Description of the specific scientific objectives or aims of this research.

There is a coming mental health crisis in response to the COVID-19 pandemic. Already, rates of depression, anxiety, and suicide are elevated in response to the pandemic (Sher, 2020; Czeisler et al., 2020). A recent study of more than 5,400 people in the US found prevalence rates of anxiety and depression in June of 2020 were 3-4 times higher than these rates in June of 2019. In this same study, 41% of respondents in June 2020 reported experiencing an adverse mental or behavioral health condition, including 13% who endorsed using substances to cope with stress or emotions and 11% who reported seriously contemplating suicide in the prior 30 days. Thus, heightened anxiety, stress, and depression will likely continue to lead to increases in self-harm and maladaptive substance use if left unchecked.

It is crucial that empirically validated psychotherapies be developed to meet these needs. These treatments must address how common barriers to treatment, including time, cost, transportation, and stigma about mental health may be exacerbated by the pandemic (Mohr et al., 2006; Brenes et al., 2015; Mohr et al., 2010). We developed a brief, virtual group-based intervention, framed as educational, Coping with Coronavirus-Related Emotions and Worries (COPING CREW), to address

these barriers. This intervention was developed over the summer by Drs. Allan, Suhr, and Austin, working with two graduate student research assistants for administration by clinical psychology graduate student therapists as part of their practicum experience at the Ohio University Psychology and Social Work Clinic (PSWC). A benefit of this approach, therefore, is that this intervention is highly scalable.

Theoretical models posit the short- and long-term stressors experienced during the pandemic exacerbate the experience of negative emotions an individual is already predisposed to experience. That is, people who are already experiencing mental health issues are going to experience more severe issues and people who may not already be experiencing mental health issues are more likely to develop mental health issues during the pandemic. Cognitive risk factors (i.e., amplifying cognitions) such as anxiety sensitivity (fear of anxiety sensations), intolerance of uncertainty (fear of the unknown), and loneliness (the subjective feeling of isolation) increase the experience of negative emotions as well as the likelihood of unhealthy coping behavior such as increased alcohol consumption or self-harm (DeMartini et al., 2011; McClelland et al., 2020).

Anxiety sensitivity, intolerance of uncertainty, and loneliness can be reduced through brief interventions applying cognitive-behavioral therapy principles (Allan et al., 2018). Reductions in these constructs lead to later reductions in anxiety, depression, and suicide (Allan et al., 2015; Albanese et al., 2018). Targeting these risk factors appears critical to address distress due to COVID-19. In 317 Ohio University faculty, staff, and students, anxiety sensitivity, intolerance of uncertainty, and loneliness accounted for 63%-75% of the variance in ratings of anxiety, depression, and suicidality as well as 49% of COVID-related disability. These findings highlight the prominent role these risk factors play in the distress many individuals are experiencing due to COVID-19. We have developed a virtual group-based intervention targeting risk factors that amplify the experience of emotional distress; the next steps in developing a scientifically rigorous intervention that can be disseminated broadly is to develop and pilot test this intervention.

The goals specific to this project are to 1) engage stakeholders in the design of a brief group-based treatment to improve COPING CREW and 2) to conduct a Stage I pilot trial through evaluating the acceptability and feasibility of COPING CREW.

Aim 1: Refine a prototype for COPING CREW by working virtually with a community partner stakeholder advisory group (SAG) as well as participants receiving the intervention. We will develop a SAG comprising one participant from each community partner. We will meet with the SAG at least three times during the 1-year project. Meetings will occur prior to starting COPING CREW groups, after running the first two groups, and after all four groups have been completed. We anticipate that building a strong relationship with this SAG will increase treatment relevance to clientele, treatment engagement, and treatment dissemination. In turn, this will enhance the durable impacts and sustainability of COPING CREW. In addition to SAGs, we will also utilize a systematic approach to engage with participant stakeholders by asking for feedback about the intervention (see Exit Interview; Appendix A.2). We have used these intervention modules separately a number of times but have yet to examine these modules as part of the same intervention. Thus, participants will provide important information on how to tailor the intervention, including the order of the components, the amount of homework specific to each component, and the length of time before the booster session to best meet their needs. Although we are confident that COPING CREW will largely be viewed as acceptable, it can be modified to increase acceptability by effective engagement with our community partners. Solicitation of feedback will occur via two mechanisms. First, information related to adaptation will be obtained from quantitative and qualitative feedback during the intervention. Second, information related to adaptation and engagement will also be collected in separate, focused feedback sessions with COPING CREW participants.

Aim 2: Examine acceptability and feasibility of COPING CREW, delivered virtually. A pilot one arm trial will be conducted, delivering the intervention to 24 participants across 4 groups.

H1: It is hypothesized that the intervention will demonstrate high acceptability and feasibility (as measured by completion rates and favorable ratings of satisfaction) across intervention modules and components within sessions. Components will include psychoeducation, challenging cognitive biases or mythbusting, behavioral exposure, behavioral experiments, and ecological momentary intervention (EMI) for treatment monitoring and homework tracking.

Description of the procedure(s) that will be performed/allowed with human participants.

FOR AIM 1:
To refine the COPING CREW prototype, feedback will be solicited from stakeholders (i.e., community partners) via three virtual meetings. Four total SAG members will participate in the meetings (compensated $40 per person per meeting). These meetings will be video recorded to ensure all stakeholder feedback is integrated into COPING CREW. The Initial meeting will involve discussion of informed consent. Informed consent will be obtained. Each meeting will involve a group-based discussion of the design, acceptability, and feasibility of the COPING CREW intervention. During these meetings they will discuss a prototype of the COPING CREW intervention. The COPING CREW prototype was developed using previous cognitive-behavioral therapy (CBT)-based AS and IU interventions (Oglesby et al., 2017; Schmidt et al., 2014, 2017) and interventions developed through the PSWC by Drs. Allan, Suhr, and Austin. In line with previous AS interventions, COPING CREW will consist of providing psychoeducation (e.g., defining common terms like anxiety), myth busting popular misconceptions clients may have about anxiety symptoms, and completing exposure exercises. The SAG meetings will involve semi-structured interviews focused on improving intervention delivery, treatment engagement, methods of advertising, and improving treatment effectiveness. SAG members will answer a series of open-ended questions (see attached feedback interview) as a group to refine the intervention.

FOR AIM 2:
The COPING CREW virtual intervention will be run through the PSWC free of charge to the participants as a pilot clinical trial. This intervention will be billed at $50 to offer an affordable treatment option and because billing is required for the PSWC. However, for participants in this study, grant funding will be utilized to cover this cost of treatment. If participants are currently engaged in treatment (either at the PSWC or elsewhere), they will not have to stop the treatments they are

receiving as our virtual intervention can augment current mental health treatment well. Interested participants who meet the screening criteria will be assigned to receive COPING CREW. First, following informed consent, eligible participants will complete a baseline assessment appointment in the week prior to the group beginning. During the baseline appointment, participants will provide informed consent, be instructed in the use of Microsoft Teams, complete a battery of self-report measures, be given a semi-structured diagnostic interview, and follow instructions to install a mobile app (mEMA) that will be used to track their mood and homework. Then, 4 groups of COPING CREW, with 6 participants per group, will be run (see timeline). Two advanced practicum graduate students in the doctoral program in clinical psychology will deliver these interventions as a team (supervised by Drs. Allan and Austin). They will work with second-year graduate students who will assist clients in completing informed consent, navigating Microsoft Teams, and installation of the mobile app that will be used to monitor intervention progress and deliver additional as-needed intervention content. This approach is consistent with the approach utilized by Drs. Allan and Austin in supervising the Brief CBT virtual practicum being offered in the PSWC.
Participants will complete four weekly 60-minute virtual intervention sessions followed by a booster session two weeks later (i.e., participants receive five sessions over the course of six weeks). Participants will complete daily surveys and homework assignments on their mobile devices. Links to follow-up surveys will be sent to participants at 1- and 3-month follow-ups with contact calls placed by research assistants to remind participants of these surveys the day the surveys arrive.

During intervention sessions, participants will receive psychoeducation, challenge cognitive biases or mythbust, and participate in behavioral exposure and behavioral experiments. An ecological momentary intervention (EMI) component of this treatment will be included for the purpose of treatment monitoring and homework tracking. Homework will be delivered and tracked via mobile app. The EMI component of COPING CREW will coincide with the timeline for virtual sessions. Participation in this EMI component involves the daily report of levels of anxiety, depression, stress, and loneliness. When participants endorse elevated levels on any of these constructs, they will receive a targeted message reminding them of the topics covered in COPING CREW. One month after the intervention session, COPING CREW participants will complete follow-up questionnaires and interview. Participants will also participate in a feedback session 3 months after the intervention.

During the EMI portion of the COPING CREW intervention, participants will complete brief questionnaires four times per day. One questionnaire will be completed at the same time every morning (based on the participant's wake time). The remaining 3 questionnaires will be administered 3 hours apart. The first of the afternoon surveys will be administered a minimum of 3 hours after the morning survey. To aid in selecting the window of questionnaire administration, participants will indicate a 12-hour block of time during which survey completion would be most convenient. The questionnaires will only be sent within this block of time. The EMI portion of the study will be completed via the Metric Wire application, a mobile app available through metricwire.com. Metric Wire is a web and mobile system designed specifically for researchers and clinical teams to collect data from participants as they go about their daily lives. Data are collected from smartphones and sent to a secure server where the data can be downloaded. The Metric Wire system is HIPAA compliant and functions on both Apple and Android smartphones. All data collected from the mobile app is encrypted before being pushed to cloud-based storage databases. Access to these databases is gated so that entry is only permitted by users the PI has granted access to. The main servers are located in the U.S. Regarding security, the data are encrypted end-to-end during transmission using TLS (1.2 &1.3) Protocol.

Participants will complete follow-up assessments 1 and 3 months after they complete the intervention. In this follow-up, they will complete questionnaires asking about their perceptions of the intervention (i.e., completing the CSQ-8) and current psychopathology (i.e., completing the same scales they did at baseline and post-intervention, see the instruments section). See below for the study flow.

Study Flow:
Study Introduction Session: Participants will meet virtually with a researcher to complete the informed consent document, fill out a W9/W8 form for compensation purposes, set up Microsoft Teams, set up the mEMA application, be given a semi-structured diagnostic interview, and complete the self report measures (specified in the measures section).

Intervention Sessions: Participants will meet virtually with PSWC therapists to complete four weekly 60-minute virtual intervention sessions followed by a booster session two weeks later (i.e., participants receive five sessions over the course of six weeks).

EMA: For the duration of the intervention sessions, participants will complete brief questionnaires (see measures section) four times per day.
One questionnaire will be completed at the same time every morning (based on the participant's wake time). The remaining 3 questionnaires will be administered at random times occurring a minimum of 90 minutes apart within three three-hour periods (also specified based on participant schedules).

EMI: Should participants endorse levels of anxiety, loneliness, or depression 50% or greater on a 0-100 scale, they will receive a brief message reminding them of the content covered in the COPING CREW intervention.

Homework: Participants will also be able to complete behavioral activation and behavioral exposure exercises agreed to by the clinician and the client and track them using the mobile app.

Follow-up Session: One and three months following the intervention session, participants will complete the same series of questionnaires that they completed during the study introduction session and following the sessions. Participants will also respond to a feedback interview regarding the acceptability of the intervention.

Description of any potential risk(s) or discomfort(s) of participation and the steps that will be taken to minimize them.

Some participants may experience slight psychological discomfort when answering questions about their mental health when responding to the questionnaires. We are using well-validated questionnaires that have previously been used in numerous research studies. Some participants may experience slight psychological discomfort when completing the interoceptive exposure exercises in the intervention. Interoceptive exposure exercises are commonly used in treatment of multiple psychiatric conditions (Boettcher et al., 2016); thus, the risk of discomfort is no greater than in routine psychological treatment. Also, participants are sharing information in a group setting in the COPING CREW sessions. Although group norms regarding confidentiality are discussed with the groups, confidentiality by other group members cannot be guaranteed. Participants will be provided with mental health resources (see attached) should they want more information or further treatment.

Additionally, there are confidentiality risks associated with being video recorded during the intervention. However, efforts have been made to decrease these risks. The intervention and all SAG meetings will be conducted via Microsoft Teams, which is a HIPAA compliant program, to ensure security of digital files. All video recordings will be stored on Microsoft Stream. Only authorized members of the research team will have access to the video files, and the files will be deleted at the end of the study.

Importantly, for all study data, confidentiality will be protected by using subject identification numbers, rather than identifying information. Research assistants will receive training on the importance of and how to ensure confidentiality. Participants will also be provided with the email address and office telephone number of the principal investigator, should they have questions or concerns.

Description of the anticipated benefits to the individual participants.

Participants completing the intervention will receive COPING CREW free of charge. Given this intervention is designed to target risk factors that amplify the experience of emotional distress, it is likely that individuals participating in this study will experience a reduction in emotional distress.

Description of the anticipated benefit(s) to society and/or the scientific community in lay language.

This study is designed to develop the first brief intervention aiming to adapt and test a brief group-based treatment to improve coping in response to COVID-19-related stressors. This treatment aims to target risk factors that amplify the experience of emotional distress, or risk factors for a variety of mental health conditions that have a substantial impact on public health. Once developed, COPING CREW will be used in prevention efforts among individuals at risk for developing disorders associated with emotional distress.

Uploaded File(s)

- Coping Crew - Therapist - 1 intro (3).pptx
- Coping Crew - Therapist - 2 Stress Sensitivity.pptx
- Coping Crew - Therapist - 3 IU (1).pptx
- Coping Crew - Therapist - 4 Loneliness (1).pptx
- Coping Crew - Therapist - 5 Booster (1).pptx
- Resources (2).docx

Confidentiality

✔	Data will be recorded with a code replacing identifiers, and a master list connecting the code and the identifier will be used.
✔	The nature of the data makes it potentially identifiable (e.g., audio or video recording, photographs).

How/where the code list will be securely stored (e.g. locked cabinet, password protected) as well as the approximate month and year it will be destroyed.

All participants will generate their own unique ID, based on the first initial of their mother's name, the first initial of their father's name, the first initial of their birthplace, their month of birth, and their date of birth. This will be done so that researchers can match deidentified data across studies. No identifiable information will be included in any analyses. The electronic master list containing the participants' subject IDs to any identifying information will be encrypted such that it cannot be opened without the password. The master list will also only be able to be opened on password protected computers. The master list will be destroyed after study completion (by or before February, 2022).

YES	NO	
✔		**Will participants be audio or video recorded?**

		✔ **Video Recorded**<br><br>**Description how/where recordings will be stored and who will have access to them, and provide an approximate month and year they will be destroyed.**<br><br>Participants who receive the intervention will be video recorded. Feedback interview sessions will also be video recorded. The video recordings will be stored in Microsoft Stream through the policies in place at the PSWC, which is a HIPAA-compliant facility. Once stored on Microsoft Stream, the video recording will be shared with authorized members of the research team. Expected date of destruction: by or before February, 2022.<br><br>Participants who participate in the SAG will also be video recorded. The video recordings will be stored in Microsoft Stream, which is a HIPAA-compliant program. Stream also meets General Data Protection Regulation privacy requirements. The recordings are private for the recorder, who will only share the recordings with members of the research team. Only authorized members of the research team will have access to this data. Expected date of destruction: by or before February, 2022.

Additional Details

Participants are sharing information in a group setting in the COPING CREW sessions. Although group norms regarding confidentiality are discussed with the groups, confidentiality by other group members cannot be guaranteed.

Compensation

YES	NO	
	✔	**Will participants receive a gift or token of appreciation?**
✔		**Will participants receive services, treatment or supplies that have a monetary value?**<br><br>**List of items and the approximate value of each.**<br>Participants who receive the intervention through the PSWC will receive the intervention free of charge. Similar interventions in the OU PSWC are offered for $50.
	✔	**Will participants receive course credit?**
✔		**Will participants receive monetary compensation (including gift cards)?**<br><br>**Detail of the amount per session and total compensation possible.**<br>Participants who receive the intervention will be compensated $40 at baseline, $40 at post-intervention, $20 at 1-month follow-up, and $30 at the 3-month follow-up (total compensation possible = $130 per participant). If participants choose to discontinue participation at any point, they will be compensated for attended sessions up to the point of discontinuation, even partially attended sessions or sessions where they did not complete all the measures.<br><br>SAG members will be compensated $40 per meeting (3 total meetings; total compensation possible = $120 per participant).
✔		**Will University funds be used to pay or otherwise compensate participants?**<br><br>**List of what participant information you need to provide to the Finance Office to document payment in the consent form.**<br>Participants will fill out a W9/W8 form that will be submitted to the OU Finance Office. This information is used for payment and tax purposes and is not saved with data. None of this data will be kept or associated with data by this lab.

Instruments & Data Analysis

Instruments

List of all questionnaires, instruments, and standardized tests.

The following questionnaires will be completed at baseline:

Demographics Questionnaire: 18-item investigator designed questionnaire used to collect basic demographic information (e.g., sex, age, ethnicity, socioeconomic status).

Investigator-created questions assessing demographic information related to the COVID-19 pandemic will be administered.

The Mini International Neuropsychiatric Interview - The M.I.N.I. 7.0.2 (8/8/16 version) (Sheehan et al., 1998) is a semistructured clinical interview that targets DSM-5 psychiatric diagnosis. For this study screener will be used to target those with psychotic features and severe suicidality and those participants will be excluded. The psychosis module will be conducted if participants endorse any of the psychotic feature screener items.

The Anxiety, Stress, and Depression Short Forms (PROMIS Anxiety, PROMIS Psychological Stress, PROMIS Depression; Cella et al., 2010) of the PROMIS Profile-29 will be used to measure anxiety, stress and depression symptoms. The PROMIS Anxiety scale measures anxiety broadly, including items to assess fear, anxious misery, hyperarousal, and somatic symptoms. Items are on a 5-point scale from 1 (Never) to 5 (Always). The PROMIS Depression scale measures depression. Items are on a 5-point scale from 1 (Never) to 5 (Always). The PROMIS Psychological Stress scale measures feelings about self and the world in the context of challenges. Items are on a 5-point scale from 1 (Never) to 5 (Always).

Anxiety Sensitivity Index 3 (ASI-3 Taylor et al., 2007) is an 18-item self-report measure of AS. This scale was developed to provide a more stable measure of the three most widely recognized AS subfactors (cognitive, social and physical concerns) than the previous ASIs provided. The measure has shown good psychometric properties (Taylor et al., 2007). The ASI-3 will be utilized to assess level of overall AS.

Intolerance of Uncertainty Scale Short Form (IUS-12: Carleton, Norton, & Asmundson, 2007). 12-items scale for measuring trait IU. In other words, it is used for assessing the degree to which individuals are able to tolerate the uncertainty of ambiguous situations, the cognitive and behavioral responses to uncertainty, perceived implications of uncertainty, and attempts to control the future.

COVID-19 Impact Battery: Three investigator-created questionnaires (CIB Behavior, CIB Worry, CIB disability; Schmidt et al., 2020 under review measure) will be used to assess COVID-19-related worries, behaviors, and difficulties.

Insomnia Severity Index (ISI; Bastien, Vallires, & Morin, 2001). The ISI is a seven-item self-report questionnaire designed to assess sleep difficulties (i.e., falling asleep, staying asleep, waking too early), satisfaction/dissatisfaction with sleep patterns, and/or interference with daily functioning. Participants will be asked to rate each item using a five-point Likert-type scale ranging from zero to four, with higher scores reflecting more severe sleep problems and greater dissatisfaction with sleep. The ISI has been found to have strong psychometric properties (Bastien et al., 2001).

Depressive Symptom Index - Suicidality Subscale (DSI-SS; Joiner et al. 2002). The DSI-SS is a 4-item measure of symptoms of suicide. The DSI-SS was designed to be a brief screening measure for suicide risk, and has well established cut points for clinical and research utility. The DSI-SS has demonstrated good psychometric properties across multiple samples (Ribeiro et al., 2012; von Glischinski et al., 2016).

Alcohol Use Disorder Identification Test (AUDIT; Babor et al., 1992): The AUDIT is a 10-item questionnaire that assesses alcohol consumption, drinking behavior, and alcohol-related problems. The AUDIT will be administered to assess drinking behavior.

Drug Use Disorders Identification Test (DUDIT; Berman et al., 2005): The DUDIT is an 11-item questionnaire that assesses drug consumption, drug use behavior, and drug-related problems. The DUDIT will be administered to assess drug use behavior.

Fear of Arousal Questionnaire (FAQ): The FAQ is a 12-item self-report measure of fear of arousal. Fear of arousal is defined as the fear of the physiological responses that are typical of anxiety (e.g., racing thoughts, racing heart, or throat tightening). The measure asks participants to rate each item on a 1 to 5 scale with 1 corresponding to no anxiety and 5 corresponding to extreme anxiety.

The following questionnaires will be completed during Weeks 1-5:
-ASI-3 (Taylor et al., 2007); see above for description
-IUS-12 (Carleton, Norton, & Asmundson, 2007); see above for description
-PROMIS Anxiety, Stress, and Depression scales (Cella et al., 2010); see above for description

The following questionnaires will be completed at post-intervention, and at the 1- and 3-month follow-ups.
-ASI-3 (Taylor et al., 2007); see above for description
-IUS-12 (Carleton, Norton, & Asmundson, 2007); see above for description
-PROMIS Anxiety, Stress, and Depression scales (Cella et al., 2010); see above for description
-CIB questionnaires (Schmidt et al., 2020 under review measure); see above for description
-ISI (Bastien, Vallires, & Morin, 2001); see above for description
-DSI-SS (Joiner et al. 2002); see above for description
-AUDIT (Babor et al., 1992); see above for description
-DUDIT (Berman et al., 2005); see above for description
-FAQ; see above for description

Interview:
At post-intervention, participants will provide information on how to tailor the intervention, including the order of the components, the amount of homework specific to each component, and the length of time before the booster session to best meet their needs. They will provide this information in a focused feedback session in which they will respond to an investigator-created semi-structured interview.

Client Satisfaction Questionnaire-8 (CSQ-8; Attkisson et al., 1979) is a 8-item self-report scale that assesses client satisfaction with a particular intervention/program. The CSQ-8 is completed by rating satisfaction with services on a 1-4 Likert-type scale. Good psychometric properties for the CSQ-8 have been found across studies (Attkisson et al., 1979; Kelly et al., 2018).

The following measures will be administered during SAG group meetings:

Interview:
The SAG meetings will involve investigator-created semi-structured interviews focused on improving intervention delivery, treatment engagement, methods of advertising, and improving treatment effectiveness.

CSQ-8 (Attkisson et al., 1979); see above for description

MetricWire measures:
The EMA items include several investigator designed questions asking participants about the impact of the COVID-19 pandemic, sleep quality, and in-the-moment ratings of anxiety, depression, stress, and loneliness. These are the items that participants will complete every morning. Participants will then be asked to rate in-the-moment anxiety, depression, stress, and loneliness and 3 random points throughout the day. Following week 1, participants will receive prompts about coping with anxiety, stress, uncertainty, and loneliness (see attached document for a week-by-week schedule). Following week 1, participants will also be asked to engage in interoceptive exposure exercises, Intolerance of uncertainty exercises, and loneliness exercises (see attached document for a week-by-week schedule). Participants will be asked to describe their experience of engaging in the exercises using the MetricWire app.

- Feedback interview COPING CREW shrunk SAG.pdf
- MINI 2.11.2020.pdf
- MINI screen 2.11.20.pdf
- ASI-3.docx
- IUS-12-SF.docx
- ISI.pdf
- DSI-SS.pdf
- AUDIT.pdf
- FAQ.pdf
- CIB measure.docx
- Feedback interview COPING CREW shrunk.pdf
- CSQ-8.pdf
- DUDIT 1.png
- DUDIT 2.png
- CIB-SF.docx
- EMA Items COPING CREW.docx
- COVID-19 Items.docx
- Demographic Questionnaire Intervention.doc
- PROMIS Anxiety-SF.pdf
- PROMIS Depression-SF.pdf
- PROMIS Psychological Stress-SF.pdf

Data Analysis

Data analysis and statistical procedures.

Acceptability/Feasibility:
COPING CREW will demonstrate acceptability and feasibility as measured by completion rates and favorable ratings of satisfaction. As one metric, the interventions will be considered acceptable and feasible if > 80% of those who initiate the baseline session complete all intervention sessions. The interventions will also be considered acceptable and feasible if > 70% of the symptom tracking sessions are completed by > 80% of participants. Finally, an average CSQ-8 general satisfaction score of > 5 (on a 7-point scale) and an average CSQ-8 component rating (i.e., intervention session, EMI) of > 5 (on a 6-point scale) will provide support for the acceptability and feasibility of these interventions and will be used as evidence that this intervention can be delivered as intended.

Power:
As this is a pilot trial, we did not conduct a formal power analysis when selecting the sample size. Rather, we used stepped rules of thumb developed to optimize the overall sample size for the pilot and main effects study (Bell et al, 2018). Although

we will not conduct formal significance tests due to the unstable nature of effect sizes from pilot studies (Leon et al, 2011), we will examine confidence intervals around our effects to inform our future fully powered RCT.

Informed Consent

Informed Consent

Obtaining signed consent for this study.

Obtaining consent without signature for the following reason:

Not practical (online or phone study)

Consent Forms

- COPING CREW Participant Consent March 2021 (1).docx
- COPING CREW Participant Screening Consent Form (3) (1).docx
- SAG Consent Form March 2021 (1).docx

How and where will the consent process occur? Will participants have an opportunity to ask questions and have them answered? What steps will be taken to avoid coercion or undue influence?

FOR AIM 1:
Consent with signature will be obtained for participation in the SAG meetings. During the initial meeting participants will meet with a researcher via Microsoft Teams to review the informed consent form and discuss any questions they may have. The informed consent process will occur via Qualtrics. Once the researcher provides an overview of the study, participants will be instructed to read the consent form in full. Once participants have read the consent form and all of their questions have been answered, those who consent will provide a digital signature on the consent document to acknowledge that they have read and understood the study's procedures, risks and benefits, and have had all questions answered. Participants will also be informed that they will have the opportunity to withdraw from the study at any time without fear of penalty.

FOR AIM 2:
The screening consent process will occur prior to the first study session and will NOT involve signed consent. Should a participant call Factors of Emotional/Affective Risk Lab to express interest in participating in this study they will be read the screening consent document (see attached) over the telephone; their verbal assent will allow the screener to occur. Should a participant contact the Factors of Emotional/Affective Risk Lab by email, they will complete the screening online and read the screening consent document themselves; continuation to the online screening questionnaire will be taken as assent. Screening survey responses will be downloaded on a weekly basis. Once the screening survey responses are downloaded, all responses on the online survey will be deleted. After participants are scheduled for the first study session or deemed ineligible for participation any identifying information will be deleted from the screening survey dataset.

Consent with signature will be obtained for participation in the intervention. This will occur during a virtual meeting with a researcher during the study introduction session. The participants will be given basic study information aloud (e.g., structure of the intervention, risks), but will be instructed to read the informed consent document on their own, with a researcher present to answer questions. The informed consent process will occur via Qualtrics. Participants will have an opportunity to ask any questions they have about the study at this point. Participants will not be asked to sign the document until they have had all of their concerns addressed adequately and they feel comfortable with participating in the project. Participants will be told that they can reschedule their participation for a later date if they would like to take time to think about their participation. Participants will indicate their informed consent by electronically signing the consent form.

All identifying consent forms will be stored electronically on a secured server affiliated with the OU PSWC. These consent forms will be destroyed 5 years after study completion (completion date January 2022; destruction date of January 2027).

If participants are currently receiving treatment at the PSWC from an investigator involved in this study, a different investigator will complete the consent process and deliver the intervention.

YES	NO	
✔		**Will all adult participants have the legal/cognitive capability to give informed consent?**
	✔	**Will any participants be minors (below age 18)?**
	✔	**Will participants be deceived or incompletely informed regarding any aspect of the study?**

ROUTING				
**Research Member**	**Status**	**Emails Sent**	**Next Email Send**	**Comments**
Accorso, Catherine	APPROVED	0		On 03/23/2021 5:41:12 PM ca265518 approved the IRB protocol.
Suhr, Julie	APPROVED	1	02/03/2021 10:00:00 AM	On 02/02/2021 10:33:53 AM suhr approved the IRB protocol.
